# Molecular characterization of *cryptosporidium* in children aged 0- 5 years with diarrhea in Jos, Nigeria

**DOI:** 10.11604/pamj.2016.25.253.10018

**Published:** 2016-12-21

**Authors:** Joseph Aje Anejo-Okopi, Julius Ocheme Okojokwu, Augustine Odo Ebonyi, Emeka Uba Ejeliogu, Samson Ejiji Isa, Onyemocho Audu, Edoama Edet Akpakpan, Esther Ebere Nwachukwu, Christabel Kelechi Ifokwe, Murna Ali, Patricia Lar, Stephen Oguche

**Affiliations:** 1 Department of Microbiology, University of Jos, Jos, Nigeria; 2 AIDS Prevention Initiative in Nigeria, Jos University Teaching Hospital, Jos, Nigeria; 3 Department of Pediatrics, University of Jos, Jos, Nigeria; 4 Department of Medicine, University of Jos, Jos, Nigeria; 5 Department of Epidemiology and Community Health, College of Health Sciences, Benue State University, Makurdi, Nigeria; 6 Department of Biological Science, Federal University, Otuoke, Bayelsa State, Nigeria

**Keywords:** Children, cryptosporidium, diarrhea, genotypes, molecular characterization, Nigeria, PCR-RFLP

## Abstract

**Introduction:**

*Cryptosporidium* is an important cause of diarrhea in children and immune-compromised individuals. Recent advances in molecular diagnostics have led to the discovery of subtype families that are thought to be more commonly associated with diarrhea. We aimed to isolate and characterize *Cryptosporidium* spp among children with diarrhea in Jos, Nigeria.

**Methods:**

Stool samples were collected from165 children aged 0-5 years with diarrhea. Cryptosporidium oocysts were examined by wet mount preparation, using formalin ether and a modified acid fast staining method. DNA was extracted from positive samples using QIAamp DNA stool mini kit and PCR-RFLP assay was carried out after quantification. Genotyping and phylogenetic analysis were done to determine the subtype families and their relatedness.

**Results:**

From the 165 children studied, 8 (4.8%) were infected with Cryptosporidium. PCR-RFLP assay and genotype characterization found the following *Cryptosporidium species: C. hominis* 6 (75%) and *C. parvum* 2 (25.0%), with family subtypes *Id-5, Ie-1 and IIa-1, IId-1* respectively.The most common species was *C. hominis* and the frequent subtype was *C. hominis-Id* 5 (62.5%).

**Conclusion:**

*Cryptosporidium* is not an uncommon cause of diarrhea in children, with *C. hominis* being the dominant species. Also *C. hominis* Id is the commonest sub-family subtype. Put together, zoonotic species may be an important cause of diarrhea in children aged 0-5 years in Jos, Nigeria.

## Introduction

*Cryptosporidium* is a protozoal parasite which gained prominence due to the emergence of chronic diarrhea in HIV/AIDS patients. It is also a major cause of gastroenteritis in immune-competent patients, leading to array of diseases from asymptomatic shedding to watery non-bloody diarrhea. A key feature of *Cryptosporidium* infection is its relative resistance of the oocysts to chlorination, raising concerns as public sources of water supplies, swimming pools for recreation and even foods such as vegetables can become contaminated [1, 2]. Other sources of infection are reservoirs such as pets and farm animals or persons [3]. However, most reported outbreaks of cryptosporidiosis among children were at day-care centers, and were traceable to contaminated source of water [4, 5].

About twenty Cryptosporidium species and 61 genotypes have been identified based on SSU-rRNA sequences, and five species commonly implicated in human cryptosporidiosis are: *C. hominis, C. parvum, C. felis, C. canis and C. meleagridis* [6, 7]. Amongst these species, *C. hominis* and *C. parvum* are the most common agents responsible of human infections with few exceptions where the prevalence of *C. meleagridis* infection is high [7]. Though there are variations in the distribution of *Cryptosporidium* depending on the ecology, weather and geographical location, *C. hominis* mainly infects humans, while *C. parvum* infects humans and domestic and or wild ruminants [8, 9]. The predominant species involved in disease causation in endemic areas and the mechanism(s) of transmission remain unclear. Molecular methods have enabled researchers to better understand the relationship between the environmental factors, disease transmission dynamics and public health interventions [10]. In Nigeria, the prevalence of cryptosporidiosis has been reported largely by conventional laboratory methods [5, 11–13] and only a few studies [14, 15] employed molecular techniques. However, molecular characterization data are specifically lacking in the whole of North-Central Nigeria. We therefore aimed to isolate and genotype the identified Cryptosporidium spp in stool samples of children aged 0-5 years with diarrhea in Jos, Nigeria.

## Methods

This was a cross-sectional study on 165 children aged 0-5 years old with diarrhoea. The children were consecutively recruited from the out-patients department (OPD) of the Primary health care (Child Welfare Center) Rwang Pam Stadium Road, and Our Lady of Apostle (OLA) hospital both in Jos-North local government area Plateau State. These health facilities were purposively selected based on the daily frequent clinic diarrhea reported cases. Children within aged 0-5years attending the hospitals’ OPD between July and September 2015 with complaints of gastrointestinal symptoms (diarrhea with/or vomiting) were included in the study. Diarrhea was defined as the passage of watery or loose stools three or more times within a 24-hours period. Acute diarrhea was defined as diarrhea lasting less than 14 days while persistent diarrhea was diarrhea that lingered for more than 14 days [16]. Vomiting occurred within the last 24 hours prior to the hospital visit. Each study participant had to provide one stool sample on the day of enrollment. A well-structured questionnaire was used to collect demographic information that included; name, age, sex, clinical symptoms including diarrhea and its duration, patient’s residence, type of toilet facility and method of water treatment. Children with dysentery, measles or any other form of disease and children whose caregivers could not ascertain their age were excluded from the study. Labeled sterile containers were provided to the caregivers of the children for the collection of stool samples. A total of 165 samples were collected and documented appropriately. The Fresh stool samples were transported to the laboratory immediately for analysis. Stool samples were divided into two; one portion was preserved with 10% formalin while the other part was preserved with 2.5% Potassium dichromate. Stool samples preserved were stored in 4ºC refrigerator for molecular analysis, while those stored in formalin were used for formol-ether concentration and microscopy as recommended [17].

Approval for the study was obtained from the Committee on Human Research Ethics of Plateau State Ministry of Health (MOH) and Ethics committee of OLA hospital, Jos. A written informed consent was obtained from the caregivers on behalf of the children prior to enrolment after clear explanation of the study objectives both in English and in Hausa for those who could not read or speak English language.

### Stool sample concentration and staining using Modified Ziehl-Neelsen (mZN)

The stool samples were concentrated according to the earlier described method [18]. Stool specimens were well mixed, and 5 ml of each fecal suspension was strained through wetted cheesecloth type gauze placed over a disposable paper funnel into a 15 ml conical centrifuge tube. Ten percent (10%) formalin was added through the debris on the gauze to bring the volume in the centrifuge tube to 15 ml. The samples were centrifuged at 500 x g for 10 minutes. The supernatants decanted and 10 ml of 10% formalin was added to the sediments and mixed thoroughly with wooden applicator stick. Four milliliters of ethyl acetate added and the tube was stoppered, shaken vigorously in an inverted position for 30secs, after which the stopper was carefully removed. Samples were again centrifuged at 500rpm x g for 10 minutes. The debris plugs were removed from the top of the tube by ringing the sides with an applicator stick. The top layer supernatants were decanted. A cotton-tipped applicator was used to remove debris from sides of the centrifuge tubes. The concentrated specimens were re-suspended in five drops of 10% formalin. The concentrated fecal samples were smeared on a microslide, air dried, fixed using methanol for 5mins, and stained with modified Ziehl-Neelsen (mZN) technique. The slides were stained with carbol-fuchsin, and differentiated in 1% hydrochloric acid-alcohol (70%) for 1 mins, counter-stained with 1% methylene blue for another 1 min. The stained slides were examined using X100 oil immersion magnification, and oocysts stain pink to red or deep purple against a blue background. The presence or absence of *Cryptosporidium* was recorded.

### DNA extraction and PCR amplification

The cryptosporidium genomic DNA was extracted from each fecal sample using the QIAamp^®^ DNA Stool Kit (QIAGEN Inc. Valencia, CA, USA) at the DNA LABS Ltd, Ungwar Sarki Kaduna according to the manufacturer’s instructions. The extracted DNA was quantified using spectrophotometer at wavelengths of 260 nm and stored at -20°C until PCR amplification. All mZN-positive specimens were genotyped by polymerase chain reaction-restriction fragment length polymorphism (PCR-RFLP) analysis of the COWP and SSU-rRNA genes. The PCR was performed with reverse and forward primers Cry-9 (5´-GGACTGAAATACAGGCATT ATCTTG-3´) and Cry-15 (5´-GTAGATAATGGA AGAGATTGTG-3´) which amplified a 550-bp fragment of 35 cycles. The SSU-rRNA genes were further amplified by nested PCR as described previously [19], to determine the species of *Cryptosporidium*, the nested PCR product of the 18S rRNA genes were digested using the VspI restriction enzyme (Promega, USA). The Restriction fragment length polymorphism (RFLP) analysis was used to distinguish *C. parvum* and *C. hominis* from other *cryptosporidium* species that may infect human. The generated amplicons were viewed in 1.5% agarose gel electrophoresis with ethidium bromide under Transilluminator UV Ultraviolet Light as earlier described [20].

### GP60 gene PCR amplification for *Cryptosporidium parvum* and *Cryptosporidium hominis* subgenotyping

A 60-kDa glycoprotein (GP60) gene fragment was amplified by nested PCR as previously described [19, 20]. The primary PCR was performed with forward and reverse primers (AL3531: 5´-ATAGTCTCCGCT GTATTC and AL3535: 3´-GGAAGGAACGATGTATCT using Roche Diagnostics, Mannheim, Germany for primary PCR mixtures, the PCR is hot started, and then left to run for 35 cycles. The generated amplicons were used for nested PCR using primers AL3532 (5´-TCCGCT GTATTCTCA GCC-3´ and AL3534 5´-GCAGAGGAACCAG CATC-3’) which amplified approximately 850 and 390-bp fragment respectively. PCR products were sequenced in both directions on an ABI Prism 3100 automated sequencer (Applied Biosystems, Foster City, California USA.) The generated sequences’ from all isolates were aligned and edited within the BioEdit software package v.7.0. The neighbor-joining phylogenetic tree was constructed using the BLAST program and ClustalX software *(* *http://blast.ncbi.nlm.nih.gov; ebi.ac.uk/pub/software/ Clustal Xv.2.0* *)* [21] for identification of genotypes and subtypes. The genetic distances were calculated using Kimura two-parameter model [22]. The reliability of the groupings was assessed by bootstrapping (100% bootstrap) analysis, with 1000 pseudoreplicates.

### DNA sequencing

The Secondary GP60 PCR products were purified using commercial kit (QIAquick^®^ Gel Extraction kit, Qiagen, Hilden, Germany). Sequencing of the purified PCR products was performed in an ABI 3720 genetic analyzer (Applied Biosystems, Forster City, CA) by using Big-Dye Terminator v. 3.1. The primers used to sequence were GP60-AL3532 and GP60-AL3534. DNA sequences were used to search the GenBank database for similarities using the nucleotide BLASTN tool program. ClustalW was used to compare sequences and phylogenetic tree was constructed using MEGA V.6.0 software.

### Statistical analysis

All data were subjected to statistical analysis using SPSS (Statistical Package for Social Science) version 22.0. Simple distribution of study variables, cross-tabulation, Chi square test, and graphs were carried out, and p<0.05 was considered statistically significant. DNA sequences were analyzed with the Basic Local Alignment Search Tool (BLAST), Molecular Evolutionary Genetics Analysis (MEGA) v. 6, and ClustalW V.2.0 Software.

## Results

**Figure 1:**
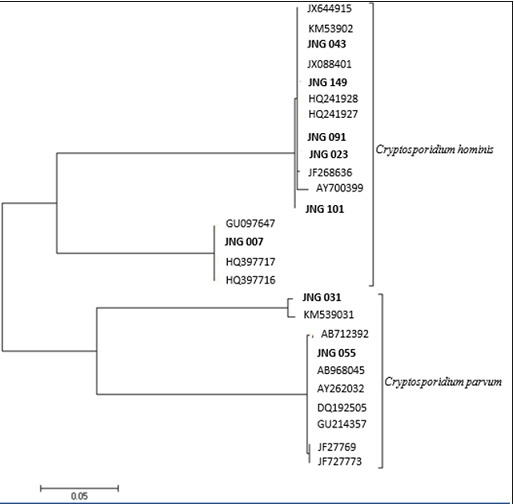
Phylogenetic analysis of *Cryptosporidium* spp (bold) by Maximum Likelihood method revealed: Id-5 (JNG 043, 149,091, 023, 101), and Ie-1 (JNG 007) of *C. hominis* subtypes and 2 of *C. parvum* subtypes (JNG 031-IIa, JNG 055-IId)

Majority of study participants were males (61.2%), females being only 38.8% and the median age was 10 months (IQR 6-22). The overall prevalence of cryptosporidiosis was 4.8%; males (4.0%) and females 6.25% (P=0.712). The prevalence of the infection was highest among aged 37- 48 months (14.3%) followed by ages 0-12 months (4.5%). The variables that were associated with a positive cryptosporidium infection include: toilet facility; pit latrine (2.5%) and bush (5.0%) versus water cistern (6.2%), p=0.033, keeping animals at home; Yes (8.7%) versus No (0.0%), p=0.001, methods of water treatment; no water treatment (7.0%) versus chemically treated water (12.5%), p=0.001. There was no significant association with type of residence, place of grazing, dwelling place of animals, contact with animal dung and source of drinking water (Table 1). The 18S rRNA PCR-RFLP analysis was performed for species identification of *Cryptosporidium spp* for all isolates. The restriction enzyme *Ssp*I was used to digest the PCR secondary product of *Cryptosporidium* spp. isolates, and this showed secondary PCR amplicons. The detected restriction digests were 450bp, 260 bp, 111 bp and 108 bp. The genotypic analysis showed phylogenetic relatedness of the *Cryptosporidium* isolates in relation to *Cryptosporidium* sequences obtained from the GenBank. The 8 clinical isolates subtyping showed; 6 *C. hominis*, and 2 *C. parvum* subtypes (Figure 1). DNA sequencing of the gp60 gene has shown extensive genetic heterogeneity among *C. hominis* and *C. parvum* isolated from children with several subtype families in both species (Id-5 and Ie-1) for C. hominis and IIa and IId for C. parvum. The alignment to the reference strains showed *C. hominis* 6(3.65%) 6/165and *C. parvum* 2(1.21%) 2/165 (Table 2). This revealed high prevalence of *C. hominis* compared to *C. parvum* subtypes in the study participants. However the *C. hominis*Ie subtype was the genetically most diverse, while the *C. hominis* (Id) subtype comprised of 5 one single subtype more related. The observed heterogeneity among the *C. hominis* and *C. parvum* subtype families may have resulted in the discriminatory power used for the subtyping of the gp60 gene (Figure 1).

**Table 1:** Socio-demographic characteristics of children with diarrhea infected with *Cryptosporidium*

Variable	No. Sample	No Positive (%)	*P*-value
**Age (months)**			0.095†
0 – 12	88	4 (4.5)	
13 – 24	17	0 (0.0)	
25 – 36	28	0 (0.0)	
37 – 48	28	4 (14.3)	
≥49	4	0 (0.0)	
**Sex**			
Male	101	4 (4.0)	0.712#
Female	64	4 (6.3)	
**Residence**			0.107#
Rural	116	6 (5.2)	
Urban	49	2 (4.1)	
**Toilet facility**			0.033+
Pit latrine	36	2 (2.5)	
Water cistern	81	5 (6.2)	
Bush	20	1 (5.0)	
No response	28	0 (0.0)	
**Animals at home**			0.001++#
Yes	92	8 (8.7)	
No	73	0 (0.0)	
**Place of animal grazing**			0.590#
Home	8	0 (0.0)	
Field	69	8 (11.6)	
**Dwelling place of animal**			1.000#
Farm	157	8 (5.1)	
Home	8	0 (0.0)	
**Contact with animal dung**			1.000#
Yes	8	0 (0.0)	
No	157	8 (5.1)	
**Source of water**			0.319†
Well	49	4 (8.2)	
Borehole	20	0 (0.0)	
Tap	96	4 (4.2)	
**Method of water treatment**			0.001++
No treatment	85	6 (7.0)	
Boiling	60	0 (0.0)	
Chemical	16	2 (12.5)	
Sieving	4	0 (0.0)	

**Key:**

# = Fisher’s exact test

† = χ^2^Chi square

++ = Very significant

**Table 2:** Cryptosporidium species and genotypes isolated from diarrheic children

Cryptosporidium Species	Number (%) positive and Subtype family
*C. hominis*	**6 (3.3)** JNG043-Id JNG149-Id JNG091-Id JNG023-Id JNG101-Id JNG007-Ie
*C. parvum*	**2 (1.21)**
	JNG 031-IIa
	JNG055-IId

**Key**: JNG Isolates identity, **Subtype family**identity:Id(5/6), Ie(1/6), IIa(1/2), IId(1/2)

## Discussion

This study found that the prevalence rate of *Cryptosporidium* in children with diarrhea in Jos was (4.8%). This prevalence was similar to earlier reported findings among children in others parts of the world with prevalence range of 40.0-4.4% [23]. The prevalence rate reported in China was 3.6% [24]. Our study prevalence rate also agrees with the reported prevalence rate of 5.9% in Uganda [25] but in sharp variance to other reported prevalence (15.6% - 19.6%) in Nigerian [11]. The low prevalence rate in our study may suggest an improved living standard in terms of sanitary conditions of the study participants, failure to detect *Cryptosporidium* oocysts in the stool of infected person which may be due to seasonal variation and intermittent nature of excretion of this parasite in stool [26]. The observed difference in the prevalence in this study could also be as a result of non-homogeneous distribution of the *Cryptosporidium* oocysts in stool specimens which could affect the detection rate of the protozoon. Despite its wide distribution in the environment, there is very little awareness about this protozoan parasite and the poor laboratory techniques which are limited to direct smear in the local medical laboratories in hospitals and private laboratories in Jos could also have accounted for this low prevalence rate. Our study lower prevalence of 4.8% falls within the lower range of the 5-10% reported worldwide in the study by Vandenberg *et al* [27] and this may be attributed to our small sample size, and the differences in the age of our study population, environmental factors (water source, sanitary condition), study timing, children nutritional status, parents socioeconomic status, undiagnosed co-infection, microscopic methods amongst other risk factors, which we did not study [28, 29].However, the disease transmission and other geographic features of *Cryptosporidium* infection among children in North-Central Nigeria are yet to be well investigated and this has contributed to paucity of data in population-based study. This study observed disproportionately high prevalence of *Cryptosporidium* infection among children aged 37-48 months (14.3%), and low (4.5%) in those aged 0-12 months. A probable reason for the higher prevalence in the former age group may be that most children at this age are just learning to be independent and are therefore, more vulnerable to acquiring the infection due to unhygienic behaviors. Gender distribution of positive *Cryptosporidium* cases were about similar (females, 6.3% versus males, 4.8%) was consistent with previous studies in Gaza [30] and Kenya [31]. This could be attributed to the fact that U-5 children of both sexes engage in almost the same recreational activities and so are likely to be equally exposed to similar environmental conditions. Our study was in contrast to an earlier study [32] which showed a wide difference in gender distribution, of males (55.6%) and females (44.4%), and the authors suggested that this difference may be due to sample size and method of detection.

The high prevalence among patients from the rural settings in our study may be attributed to low socio-economic status that is known to be associated with unhygienic conditions such as close contact with domestic animals, poor food preparations and storage, poor housing and household overcrowding [33]. In most developing countries like Nigeria, cryptosporidium infection was common among children aged ≥6 months and decreases with older age. It is speculated that breast-feeding confers some form of protection, which may be through mother-child immunoglobin response coupled with not using contaminated water. This may suggest why *Cryptosporidium* infection is mostly delayed till six months and beyond when complementary foods are introduced. However, the infection is common in children but tends to decrease with increasing age which suggests development of immunity from frequent exposure to infective agents in the environment.

The study observed a high frequency of *Cryptosporidium* infection among rural residents but found no significant association. This can be attributed to increased exposure to zoonotic infection, unsanitary environments and close contact with soil contaminated by animal feces. An earlier study also reported that residing in rural areas appears to be a contributing factor to increase of *Cryptosporidium* infection risk [34].*Cryptosporidium* infection was observed to have a significant association with the type of toilet facility, history of animals at home and methods of water treatment. This corroborates earlier studies that poor socio-economic status was associated with poor toilet facility, unhygienic animal keeping and drinking of inadequately treated water in countries that lack basic public functional facilities [35]. This suggests poor personal hygiene and an unsanitary habit of caregivers, and also showed zoonotic transmission pattern as animals have been identified as reservoir source [36]. The water treatment methods recorded highest prevalence amongst those with improper water treatment, and this was in agreement with previous studies that reported outbreaks in both adults and children due to improper water treatment [37]. *Cryptosporidium* oocysts are not eliminated by chlorination or domestic disinfectants. Generally, the treatment of water to eliminate any infestation by *Cryptosporidium* has not been completely successful. Perhaps it could be due to the presence of *Cryptosporidium* oocysts in the water, which tends to increase the chances of been infected. On the other hand, children whose parents practice treatment by boiling and sieving recorded no Cryptosporidium infection. This can be attributed to elimination of *Cryptosporidium* oocysts from the water by these methods. Thus boiling and sieving are effective methods for treatment of drinking water. The results of the study also showed that factors such as; place of grazing, dwelling of animals, contact with animal dung and water source did not have a significant association, though findings from epidemiological studies have implicated these factors as risks for transmission [38].

On molecular characterization, the study identified two *Cryptosporidium* species (*C. hominis* (6/8, 75.0%) and *C. parvum* (2/8, 25%) with different subtypes (*C. hominis* (Id-5/6, Ie-1/6) and *C. parvum* (IIa-1/2, IId-1/2). These subtypes have not yet been described in children samples in Jos. This study showed that these species are the dominant prevalent species that infect humans in the Jos area. *Cryptosporidium hominis* had more subtypes 6 (Id-5, Ie-1), and C. parvum 2 subtypes (*IIa*-1, *IId*-1). Our findings corroborates those of earlier reported studies in other parts of the world [39, 40], including Kenya [29], India [ 41], Malawi [42] and Uganda [25] that *C. hominis* are more prevalent than *C.parvum* and it’s the leading cause of human cryptosporidiosis in children studied. The human cryptosporidiosis associated with children can be traced to other known *Cryptosporidium* species; *C. canis, C. felis, C. meleagridis, C. muris,* which occasionally cause infection especially among immune-compromised individuals [30]. This may explain the reason for transmission from humans to humans and through unhygienic environment. In this study, although majority of the children had no contact with animals, the infection might likely be from human contaminated fecal materials or may be due to consumption of water contaminated by animals’ feces. The contact with animal could have been a source of infection since animals are known reservoirs for transmission. It could be argued that since *Cryptosporidium* are not host specific, infection can be from animal reservoirs to humans [37]. The observed variations in the rates of *C. hominis* and *C. parvum* may suggest differences in study location and source of infection. The presence of these species showed that transmissions in our study population could be both anthroponotic and zoonotic [43, 44]. The result of phylogenetic analysis grouped the *C. hominis* into two subtype allele (*Id* and *Ie*-1), and *C. parvum* into subtype *IIa* and *IId* which are known to be zoonotic. This suggests possible transmission from animal reservoir to humans especially in individuals that are immunosuppressed.

## Conclusion

The study showed the predominance of *C. hominis* over *C. parvum* among the diarrheic children. However, the frequency of the Cryptosporidium allele subtypes appears different from earlier findings in other parts of the world. The study also showed high prevalence of the family subtypes in the Jos area and advocates the use of molecular techniques in investigating suspected cryptosporidium infection in children with diarrhoea. There is a need for healthcare providers especially clinician and laboratory scientist to also routinely screen children with diarrhea for *Cryptosporidium* in addition to other intestinal pathogens. There is a need to improve living conditions so as to reduce the risk of children from contracting the infection especially among the rural dwellers. Furthermore, studies with larger sample sizes are needed to determine the prevailing cryptosporidium subtypes in Nigeria, to better understand possible routes of transmission, and to apply timely intervention measures to prevent spread of the disease.

### What is known about this topic

- Cryptosporidium infection is associated with diarrhea;
- Molecular characterization has also been reported elsewhere;
- Transmission associated with poor socioeconomic status has also been reported.

### What this study adds

- New data to existing data on Cryptosporidium infection among young children;
- Molecular characterization of isolates from children with diarrhea in Jos;
- Predominant Cryptosporidium subtypes identified.
